# Anti-TNF Drugs for Chronic Uveitis in Adults—A Systematic Review and Meta-Analysis of Randomized Controlled Trials

**DOI:** 10.3389/fmed.2019.00104

**Published:** 2019-05-24

**Authors:** Inês Leal, Filipe B. Rodrigues, David Cordeiro Sousa, Gonçalo S. Duarte, Vasco C. Romão, Carlos Marques-Neves, João Costa, João Eurico Fonseca

**Affiliations:** ^1^Department of Ophthalmology, Hospital de Santa Maria, Lisbon, Portugal; ^2^Faculdade de Medicina, Centro de Estudos das Ciências da Visão, Universidade de Lisboa, Lisbon, Portugal; ^3^Laboratório de Farmacologia Clínica e Terapêutica, Faculdade de Medicina, Universidade de Lisboa, Lisbon, Portugal; ^4^Clinical Pharmacology Unit, Faculdade de Medicina, Instituto de Medicina Molecular, Universidade de Lisboa, Lisbon, Portugal; ^5^Department of Rheumatology, Hospital de Santa Maria, Lisbon, Portugal; ^6^Rheumatology Research Unit, Faculdade de Medicina, Instituto de Medicina Molecular, Universidade de Lisboa, Lisbon, Portugal; ^7^Faculdade de Medicina, Centro de Medicina Baseada na Evidência, Universidade de Lisboa, Lisbon, Portugal; ^8^Cochrane Portugal, Faculdade de Medicina, Universidade de Lisboa, Lisbon, Portugal

**Keywords:** non-infectious uveitis, anti-tumor necrosis factor drugs, adalimumab, etanercept, safety, efficacy

## Abstract

**Background:** We aimed to assess efficacy and safety of anti-tumor necrosis factor (TNF) drugs for adult chronic non-infectious uveitis (NIU).

**Methods:** CENTRAL, MEDLINE, and EMBASE, were searched from inception to January 2019. Double-masked randomized placebo-controlled trials, assessing any anti-TNF vs. best medical intervention/standard of care in adults with chronic NIU were considered. The PRISMA and SAMPL guidelines were followed. The risk of bias was assessed using the Cochrane risk of bias tool. Overall quality of the evidence was assessed according to GRADE. PROSPERO registration: #CRD42016039068. The primary efficacy and safety outcomes were preservation of visual acuity (VA) and withdrawals due to adverse events, respectively. Meta-analysis of efficacy analysis was not performed due to significant clinical heterogeneity between studies' population and interventions.

**Results:** A total of 1,157 references were considered and 3 studies were included. The overall risk of bias was moderate. In active NIU, adalimumab group showed an increased likelihood of VA preservation (risk ratio (RR) 1.75, 95%CI 1.32 to 2.32, *n* = 217), whereas the etanercept group did not (RR 0.81, 95%CI 0.57 to 1.14, *n* = 20). In inactive NIU, adalimumab was associated with increased likelihood of VA preservation (RR 1.31, 95%CI 1.12 to 1.53, *n* = 226). The rate of adverse events did not differ between anti-TNF and control arms (RR 1.03, 95%CI 0.94 to 1.13, *n* = 410).

**Conclusions:** There is high quality evidence that adalimumab decreases the risk of worsening VA in active and inactive NIU and very low quality evidence that the risk of etanercept worsening VA in inactive NIU is not different from placebo. Moderate quality evidence suggests that anti-TNF agents are not different from placebo on the risk of study withdrawal.

## Introduction

### Rationale

Uveitis comprises a heterogeneous group of inflammatory diseases of the uvea of both infectious and non-infectious etiologies. Specifically, non-infectious uveitis (NIU) is thought to result from an immune-mediated response to ocular antigens (1, 2). Given its estimated incidence (52/100 000 person-years) and reported prevalence (115–121/100,000 people) (3, 4) NIU brings with it significant burden for the healthcare systems and in this working age group (2, 5). Complications of uveitis can be sight threatening, severely impairing quality of life (1, 6).

The therapeutic goal for NIU is to reduce ocular inflammation, thus preventing damage to ocular structures and consequent vision loss. Corticosteroids are the mainstay of therapy, however, these drugs are often incapable of proper inflammation control and have long-term systemic side effects (7, 8). When inflammation is not controlled by corticosteroids or side effects are intolerable, systemic immunomodulatory therapy (IMT) should be considered (5, 9). Current IMT includes the inhibition of TNF, achieved with mAb, such as infliximab, adalimumab, golimumab, and certolizumab-pegol, or with receptor fusion protein (FP), etanercept (9, 10).

Although anti-TNF drugs are approved for many other chronic immune-mediated inflammatory diseases, they are still used off-label for NIU, with the exception of adalimumab that has recently been approved for this indication (8, 11).

### Objective and Research Question

We conducted a systematic review with meta-analysis of randomized controlled trials (RCT) to summarize the available evidence, to grade the quality of the available studies, to mark out areas of intellectual disagreement, and to draw attention to matters to all stakeholders requiring future development.

## Methods

### Study Design

For a detailed description of the methods, please refer to Appendix 1.

### Systematic Review Protocol

The protocol followed the PRISMA-P guidelines (12) and it was registered at Prospero database (CRD42016039068). Reporting followed the PRISMA and SAMPL guidelines (13, 14).

### Participants, Interventions, Comparators

We analyzed parallel RCT of any duration, assessing efficacy and safety of anti-TNF vs. control interventions in patients with chronic NIU. RCT had to include adult patients with a clinical diagnosis of chronic NIU, irrespective of the etiology.

### Search Strategy

For the identification of studies considered for inclusion in this review, detailed search strategies were developed for each database explored. Please refer to Appendices 2–4 for the MEDLINE, EMBASE and CENTRAL search strategies, respectively. The search strategies for the other databases can be found in Appendices 5–8.

### Data Sources, Studies Sections, and Data Extraction

Wesearched MEDLINE, EMBASE, CENTRAL, OpenSIGLE.inist.fr,NTIS.gov, ClinicalTrials.gov, and ClinicalTrialsRegistry.eu. Gray literature was retrieved from appropriate databases. Clinical trials registries were pursued. Whenever necessary, authors of published trials were contacted for further information and unpublished data. Search strategies were designed, tested and applied to databases by one of the authors (FR).

#### Study Selection

Independent review authors assessed if the studies identified by the search strategy were eligible. The same authors independently screened the full-texts of potentially eligible studies. Disagreements were resolved by consensus, or with the participation of a third author.

### Data Analysis

#### Data Collection Process

Two authors independently assessed the full-text articles of included studies for methodological quality and data extraction, then extracted the data onto standardized forms and crosschecked them for accuracy. Disagreements were resolved by discussion, and consensus was reached with participation of three authors.

#### Risk of Bias in Individual Studies

The recommended Cochrane Collaboration's tool for assessing risk of bias was used in this review, which targets six specific risk of bias domains (15). Two additional domains were added: for-profit bias, and prospective trial registration. Two independent review authors performed critical assessments for each domain. Disagreements were resolved by discussion and, if needed, consensus was reached with the participation of a third author.

#### Summary Measures

The primary efficacy outcome was preservation of best-corrected visual acuity (VA), in logMAR, measured and presented according to the standard procedure developed for the Early Treatment Diabetic Retinopathy Study (16) in the end of the study.

The primary safety outcome was withdrawals due to adverse events.

The secondary efficacy outcomes were: change from baseline in anterior chamber and/or vitreous inflammation grade and (according to Standardization of Uveitis Nomenclature (SUN) (17, 18) recorder in the last measurement. In case one eye improved but the other eye deteriorate, we considered the changes from baseline in anterior chamber and/or vitreous inflammation reported in the worsening eye; other secondary outcomes were median time to Optical Coherence Tomography (OCT), evidence of cystoid macular oedema (CME), change from baseline in the score after 16–20 weeks of therapy obtained in a vision-specific questionnaire, the National Eye Institute Visual Functioning Questionnaire 25 (NEI-VFQ) (19).

The secondary safety outcomes were number of patients with: (i) infections, (ii) new onset or reactivated tuberculosis, (iii) injection site or allergic reactions, (iv) immunogenicity related adverse events, and (v) other adverse events.

For dichotomous outcomes, we retrieved the total number of included patients for each arm (i.e., anti-TNF and control), and the number experiencing the outcome. We reported numerators and denominators for all percentages. For continuous outcomes, we retrieved the mean or median—the latter converted to mean using statistically validated methods (20). We reported means and standard deviations (SD). For counts, we reported the mean per participant and SD as presented by the authors. We carried an intention-to-treat analysis using risk ratios for dichotomous outcomes, mean differences for continuous outcomes, and rates and SD for counts. The proportion of patients with adverse events was compared between treatment arms, and further analysis was performed in the most frequent complications reported in trials. Data was pooled from the studies where adequate, and used for comparison.

#### Synthesis of Results

Statistical analysis was performed with Review Manager v 5.3. Dichotomous data were preferentially reported in this review as risk ratios using a Mantel-Haenszel fixed-effects (FE) model and 95% confidence intervals (95% CI). Continuous outcomes, such as efficacy measures, were reported as mean differences and 95% CI. For counts, the rate ratios were pooled using the generic inverse-variance method. Where data from the studies reports could not be combined into a meta-analysis, a narrative approach to synthesis of the results was included in the review text.

Heterogeneity between trial results was tested using an *I*^2^ statistic to quantify inconsistency across studies. When considerable heterogeneity was present (i.e., *I*^2^ > 50%), the possible causes of heterogeneity were explored by conducting subgroup analysis. Where heterogeneity could not readily be explained by the exploratory analyses performed, it was incorporated into a Random-Effects (RE) meta-analysis model.

#### Risk of Bias Across Studies

Publication bias was planned to be assessed through visual inspection of funnel plots asymmetry and Peters' regression tests (21, 22), if more than 10 studies per outcome were available (15). Unfortunately we did not have enough power to perform these analyses.

#### Additional Analysis

Subgroup analysis was pre-planned for the following: uveitis etiology (primary vs. secondary; different secondary causes), risk of bias, pharmacological compound (infliximab vs. adalimumab vs. certolizumab vs. golimumab vs. etanercept), age of participants, mean follow-up time, steroid-resistant or dependent, and BCVA (15). Unfortunately, we did not have enough power to perform these analyses.

#### Confidence in Cumulative Evidence

All primary outcomes were evaluated according to quality of evidence using the GRADE working group methodology (23). Secondary safety outcomes and the proportion of patients achieving an excellent functional outcome at 90 days after randomization were also graded.

## Results

### Study Selection and Characteristics

#### Study Selection

**Figure 1:**
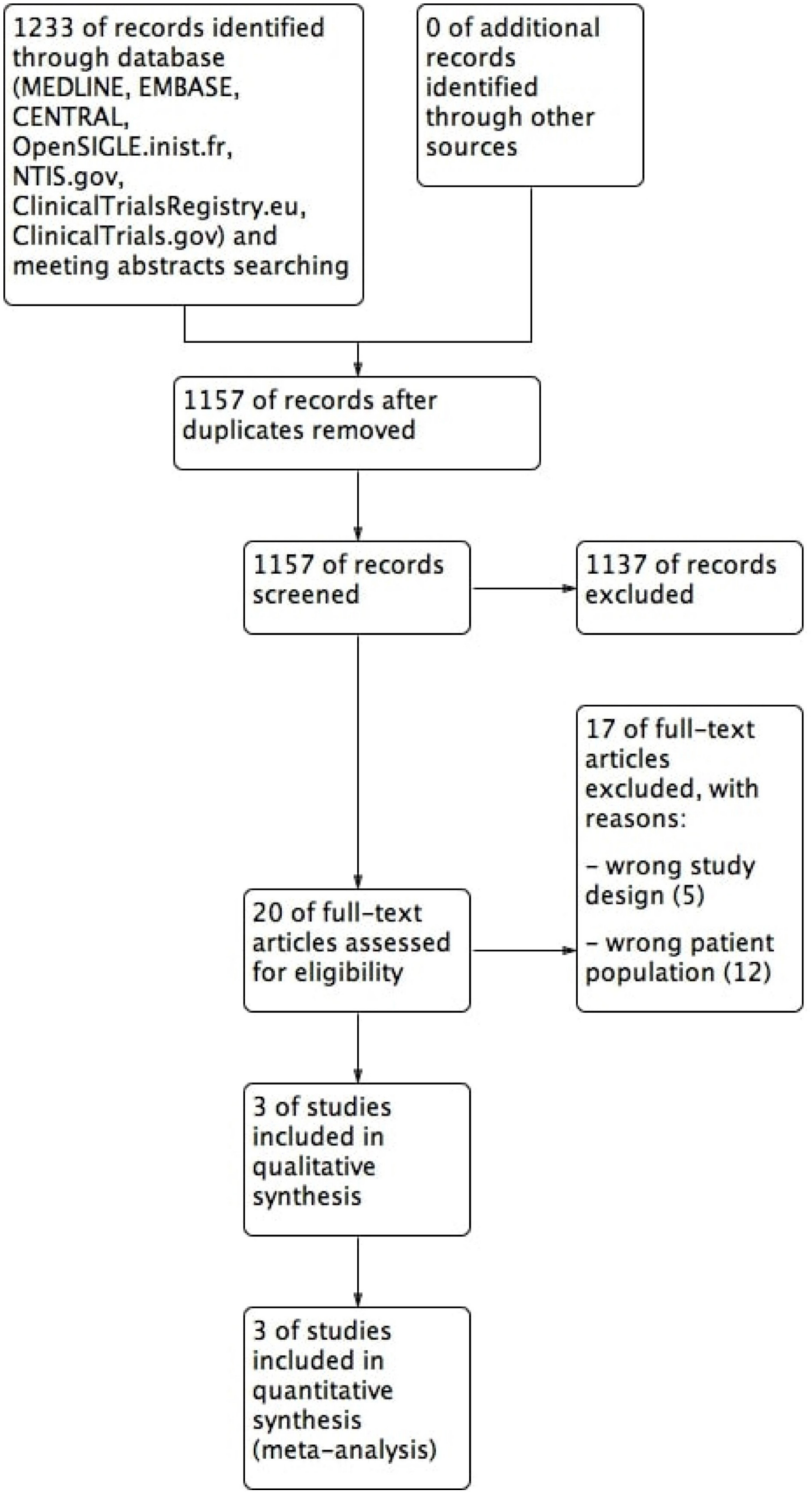
Flow diagram.

A total of 1,233 references were identified (MEDLINE: 1052; Embase: 137; CENTRAL: 44) (Figure 1). No additional records were identified through other sources or manual search. After duplicates removal, 1,157 papers were screened for title and abstract and 20 references were examined in full-text. Of these, 17 studies were excluded, 5 due to study design and 12 due to wrong patient population. Three studies were included in the final analysis: Foster et al. (24) and VISUAL I and II (25, 26).

#### Study Characteristics

Of the three studies included in our analysis, VISUAL I (25) and VISUAL II (26) assessed efficacy and safety of adalimumab in controlling inflammation in active uveitis and preventing uveitic flare in inactive uveitis, respectively, and the other study [hereby defined as Foster et al. (24)] assessed the efficacy and safety of etanercept in preventing flare of uveitis previously controlled with methotrexate. All studies compared placebo and anti-TNF between arms. VISUAL I enrolled 217 patients, VISUAL II enrolled 226 patients and Foster et al. (24) enrolled 20 patients. Overall, the studies involved 458 participants−230 in the anti-TNF arm and 228 in the control arm, based on an intention-to-treat population. VISUAL I included adult patients with a diagnose of active non-infectious intermediate uveitis, posterior uveitis or panuveitis despite the use of prednisone (10 to 60 mg/day) or an equivalent corticosteroid for 2 or more weeks before screening; VISUAL II included adult patients with a diagnosis of inactive non-infectious intermediate, posterior or panuveitis for at least 28 days before the baseline visit, and use of 10–35 mg/day of oral prednisone to maintain inactive disease; finally, in Foster et al. (24), eligible participants were adult patients treated for recurrent uveitis and taking methotrexate (≤15 mg/kg per week) for at least 12 weeks with control of uveitis. Main study characteristics are depicted in Table 1.

**Table 1:** Individual studies' characteristics.

**Study**	**Type of study**	**Population**	**Intervention**	**Control**	**Main outcomes**	**Concomitant immunosuppressive drugs**	**Pre-specified maximum duration**	**Observations**
Foster	Single-centre, double-masked, randomized, placebo-controlled phase 3 trial	Adults patients with recurrent uveitis and under a low dosage (≤0.15 mg/kg per week) of MTX for at least 12 weeks with control of uveitis	Subcutaneous etanercept 25 mg twice a week	Matched placebo administered by the same route and frequency of the intervention	Control of inflammation, VA and adverse events	No	24 weeks	All patients had a mandatory tapering of MTX at 2.5 mg/week starting at 2 weeks after the 1st dose of study medication
VISUAL I	Multi-center, double-masked, randomized, placebo-controlled phase 3 trial	Adult patients with the diagnosis of active non-infectious intermediate uveitis, posterior uveitis or panuveitis despite oral steroids for 2 or more weeks	Subcutaneous adalimumab (loading dose of 80 mg followed by a dose of 40 mg eow)		TTF occurring at or after week 6 (a multicomponent outcome that was based on assessment of new inflammatory lesions, best corrected visual acuity, anterior chamber cell grade and vitreous haze grade	Participants allowed to receive up to one conventional immunosuppressive drugs at pre-specified doses	80 weeks or when a pre-specified number of TF occurred	All patients received a mandatory oral prednisone burst followed by tapering of prednisone over the course of 15 weeks
VISUAL II	Multi-center, double-masked, randomized, placebo-controlled phase 3 trial	Adult patients with inactive, non-infectious intermediate, posterior or panuveitis uveitis controlled by 10–35 mg/day prednisone	Subcutaneous adalimumab (loading dose of 80 mg followed by a dose of 40 mg eow)		TTF (a multicomponent outcome based on assessment of new inflammatory lesions, best corrected VA, anterior chamber cell grade and vitreous haze grade)		80 weeks or when a pre-specified number of TF occurred	All patients had a mandatory prednisone taper from week 2

*MTX, methotrexate; eow, every other week; VA, visual acuity; TTF, time to treatment failure; TF, treatment failure*.

Randomization was stratified by baseline immunosuppressant treatment in VISUAL I and VISUAL II, but not in Foster et al. (24). Intention-to-treat analysis was used in all three studies to evaluate efficacy endpoints.

Mean age ranged from 42 to 48 years and sex distribution favored female in all three studies (Supplementary Table 1). However, in VISUAL I, sex distribution was not disclosed between arms. Supplementary Table 1 summarizes population baseline characteristics by individual study.

### Synthesized Findings

Table 2 summarizes the results of individual studies.

**Table 2:** Efficacy and Safety results by individual study.

	**Foster**	**VISUAL I**	**VISUAL II**
	**Anti-TNF Placebo**	**Anti-TNF Placebo**	**Anti-TNF Placebo**
Risk of not worsening BCVA risk ratio (95% CI)	0.90 (0.69−1.18)	1.75 (1.32, 2.32)	1.31 (1.12, 1.53)
Mean change in anterior chamber cell grade MD (95%CI)	N.S.	−0.29 (−0.51, −0.07)	−0.14 (−0.37, 0.08)
Mean change in vitreous haze grade (mean between group difference, 95%CI)	N.S.	−0.27 (−0.43, −0.11)	−0.13 (−0.28, 0.01)
Median time to OCT evidence of CME on or after week 6 (months)	N.S.	11.1^a^ 6.2^b^	N.S.
Change in VFQ-25 total score	N.S.	4.20 (1.02, 7.38)	2.12 (−0.84, 5.08)
Serious infections (rate ratio and 95%CI)	N.S.	1.18 (0.42, 3.27)	1.14 (0.23, 5.68)
TB (active and latent) (risk ratio and 95% CI)	N.S.	5.04 (0.24, 103.90)	2.97 (0.31, 28.17)
Injection-site events (rate ratio and 95%CI)	N.S.	2.84 (1.60, 5.05)	1.69 (1.00, 2.84)
Number of patients with anti-drug antibodies (risk ratio and 95% CI)	N.S.	7.06 (0.37, 135.16)	14.87 (0.86, 257)
Allergic adverse events (rate ratio and 95%CI)	N.S.	1.65 (0.84, 3.23)	0.47 (0.17, 1.31)
Adverse events (rate ratio and 95%CI)	1.50 (0.60, 3.74)^c^	1.08 (0.99, 1.18)	0.97 (0.89, 1.06)
Serious adverse events (rate ratio and 95%CI)	N.S.	2.12 (1.11, 4.04)	0.98 (0.47, 2.06)
Adverse events leading to death (rate ratio and 95%CI)	1.00 (0.02, 46.05)^c^	3.20 (0.13, 76,58)	4.20 (0.19, 91.13)
Events of lupus or lupus-like events (rate ratio and 95%CI)	N.S.	3.00 (0.12, 73.21)	1.00 (0.02, 50.40)
Events of demyelination (rate ratio and 95%CI)	N.S.	3.00 (0.12, 73.21)	1.00 (0.02, 50.40)
Events of cancer (rate ratio and 95%CI)	N.S.	6.40 (0.33, 125.89)	2.20 (0.08–62.81)
Total number of withdrawals (risk ratio and 95% CI)	5.00 (0.27, 92.62)	2.59 (1.13, 5.97)	0.87 (0.44, 1.69)
Withdrawals due to adverse events (risk ratio and 95% CI)	5.00 (0.27, 92.62)	3.36 (0.95, 11.90)	1.42 (0.56, 3.59)
Withdrawals due to lost to follow-up (risk ratio and 95% CI)	1.00 (0.02, 46.05)	9.08 (0.49, 166.69)	0.14 (0.01, 2.71)
Withdrawals by patient (risk ratio and 95% CI)	1.00 (0.02, 46.05)	5.04 (0.24, 103.90)	0.66 (0.11, 3.88)
Withdrawals for other reasons (risk ratio and 95% CI)	1.00 (0.02, 46.05)	1.68 (0.41, 6.87)	0.66 (0.11, 3.88)
Withdrawal due to lack of efficacy (risk ratio and 95% CI)	1.00 (0.02, 46.05)	0.50 (0.05, 5.48)	0.14 (0.01, 2.71)

*BCVA, best corrected visual acuity; CI, confidence interval; TNF, tumor necrosis factor; N.S., non-stated; OCT, optical coherence tomography; CME, cystoid macular edema; VFQ, visual function questionnaire; TB, tuberculosis; MD, mean difference*.

a *evaluated in 55 patients (CME was included only for patients who did not have CME at baseline)*.

b *evaluated in 45 patients (CME was included only for patients who did not have CME at baseline)*.

c *calculated as risk ratio*.

In VISUAL I, adalimumab was efficacious, as the risk ratio of not worsening BCVA was 1.75 (95% CI 1.32 to 2.32) comparing to placebo. Moreover, the results favored adalimumab for change in anterior chamber [mean difference between groups: −0.29 (95% CI −0.51 to −0.07)] and vitreous inflammation grades [mean difference: −0.27 (95% CI −0.43 to −0.11)], median time to OCT evidence of CME (placebo arm: 6,2 months, anti-TNF arm: 11,1 months) and change in VFQ-25 (27) composite score (placebo arm: −5.5; anti-TNF arm: −1.3).

In VISUAL II, adalimumab was efficacious, as the risk ratio for not worsening BCVA was 1.31 (95% CI 1.12 to 1.53) vs. placebo. The results for other efficacy outcomes were not different between groups: change in anterior chamber [mean difference: −0.14 (95% CI −0.37 to 0.08], vitreous inflammation [mean difference: −0.13 (95% CI −0.28 to 0.01)] grades and change in VFQ-25 composite score (placebo arm: 1.24; anti-TNF arm: 3.36). The median time to OCT evidence of CME was not estimable.

In Foster et al. (24), etanercept was not associated with a risk reduction of not worsening BCVA comparing to placebo (risk ratio 0.90, 95% CI 0.69 to 1.18).

#### Synthesis of Results

##### Efficacy

In our view, there is significant clinical heterogeneity between studies' population and intervention. Therefore, we decided not to pool the 3 studies together for efficacy analysis, as the pooled analysis would not lead to a meaningful conclusion. Results of individual studies were incorporated in Table 2 and have been described above.

##### Safety

**Figure 2:**
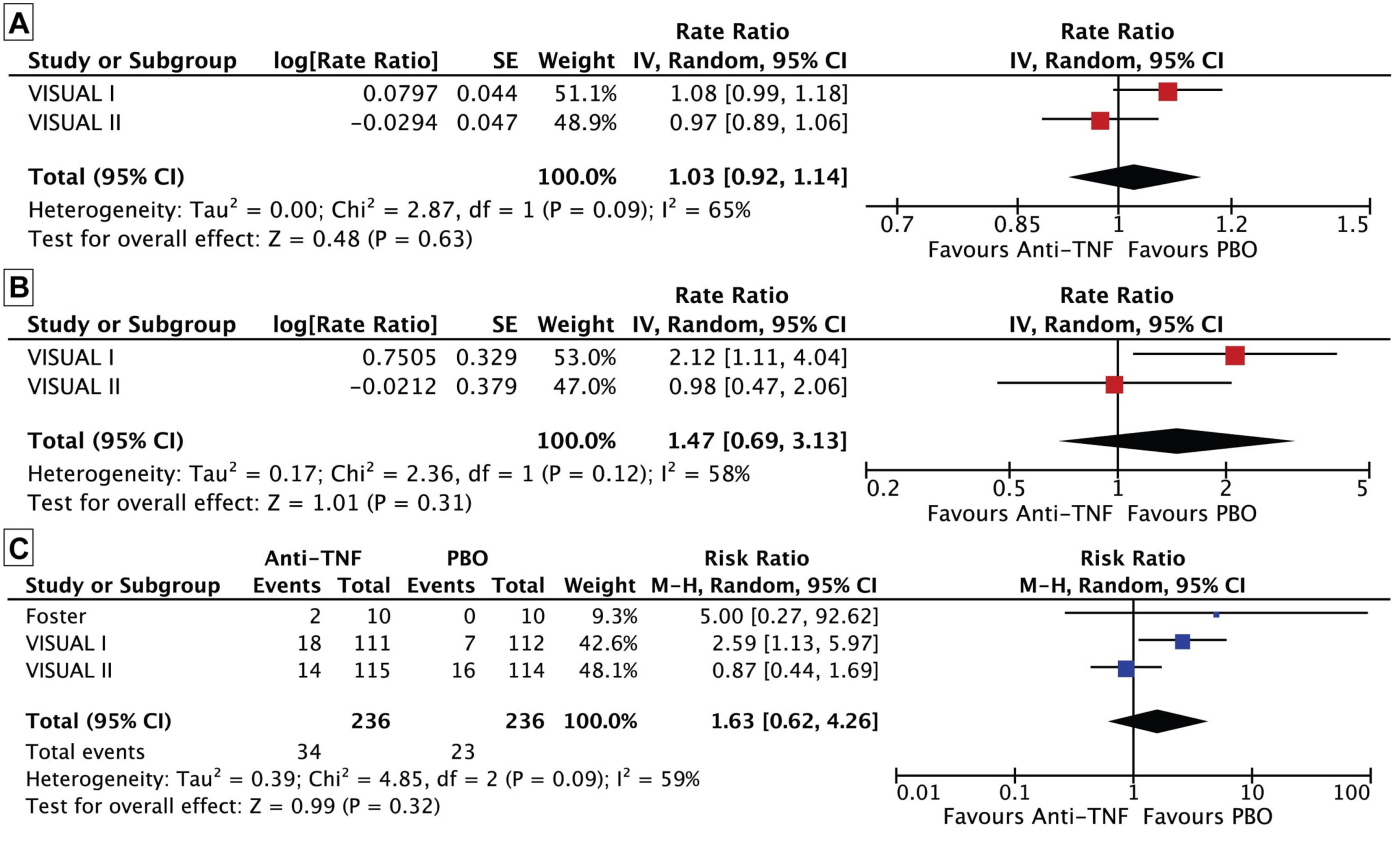
Forest plot. **(A)** Rate of adverse events between the anti-TNF and control arms. **(B)** Rate of serious adverse events between the anti-TNF and control arms. **(C)** Risk of withdrawing between the anti-TNF and control arms.

However, for safety outcomes, as all studies used anti-TNF drugs as active intervention and safety outcomes analyzed are specific to this drug class, we decided to pool and quantitatively assess them (28). The rate of adverse events in VISUAL I and II trials did not differ between the anti-TNF and control arms (rate ratio 1.03, 95% CI 0.92 to 1.14, *n* = 443, *I*^2^ = 65%; Figure 2A), nor did that of serious adverse events (rate ratio 1.47, 95% CI 0.69 to 3.13, *n* = 443, *I*^2^ = 58%; Figure 2B and Table 3).

**Table 3:** Safety results meta-analysis.

	**Anti-TNF vs. Placebo**		
	**Foster**	**VISUAL I**	**VISUAL II**
Serious infections (rate ratio and 95%CI)	N.S.	1.17 (0.49, 2.76)	
TB (active and latent) (risk ratio and 95% CI)	N.S.	3.59 (0.59, 21.81)	
Injection-site events (rate ratio and 95%CI)	N.S.	2.16 (1.29, 3.60)	
Number of patients with anti-drug antibodies (risk ratio and 95% CI)	N.S.	10.38 (1.34, 80.69)	
Allergic adverse events (rate ratio and 95%CI)	N.S.	0.94 (0.28, 3.19)	
Adverse events (rate ratio and 95%CI)	1.50 (0.60, 3.74)^a^	1.02 (0.92, 1.14)	
Serious adverse events (rate ratio and 95%CI)	N.S.	1.47 (0.69, 3.13)	
Adverse events leading to death (rate ratio and 95%CI)	1.00 (0.02, 46.05)^a^	3.68 (0.40, 33.55)	
Events of lupus or lupus-like events (rate ratio and 95%CI)	N.S.	1.94 (0.16, 23.02)	
Events of demyelination (rate ratio and 95%CI)	N.S.	1.94 (0.16, 23.02)	
Events of cancer (rate ratio and 95%CI)	N.S.	3.99 (0.43, 37.03)	
Total number of withdrawals (risk ratio and 95% CI)	1.63 (0.62, 4.26)		
Withdrawals due to adverse events (risk ratio and 95% CI)	2.04 (0.99, 4.21)		
Withdrawals due to lost to follow-up (risk ratio and 95% CI)	1.00 (0.02, 46.05)	1.14 (0.02, 67.56)	
Withdrawals by patient (risk ratio and 95% CI)	1.00 (0.02, 46.05)	1.25 (0.19, 8.18)	
Withdrawals for other reasons (risk ratio and 95% CI)	1.00 (0.02, 46.05)	1.17 (0.39, 3.52)	
Withdrawal due to lack of efficacy (risk ratio and 95% CI)	1.00 (0.02, 46.05)	0.31 (0.05, 1.95)	

BCVA, best corrected visual acuity; CI, confidence interval; TNF, tumor necrosis factor; N.S., non-available; OCT, optical coherence tomography; CME, cystoid macular edema; VFQ, visual function questionnaire; TB, tuberculosis;

a *calculated as risk ratio*.

Regarding safety outcomes, withdrawals were pooled together for the three studies, and there were no differences in the risk of withdrawing in the anti-TNF arm group comparing to placebo (risk ratio 1.63, 95% CI 0.62 to 4.26, *n* = 472, *I*^2^ = 59%) (Figure 2C). Some other safety outcomes were only pooled together for both VISUAL studies due to absence of correspondent information in Foster et al. (24). In both VISUAL studies, the results (Table 3) significantly favored placebo for injection site-events (rate ratio: 2.16, 95%CI 1.2 to 3.60, *n* = 443) and number of patients with anti-drug antibodies (risk ratio: 10.38, 95%CI 1.34 to 80.69, *n* = 443). There were no differences in rate or risk ratios for the other analyzed endpoints between adalimumab and placebo arms. In Foster et al. (24), the results for the risk of patients experiencing adverse effects were also not different (risk ratio 1.50, 95% CI 0.60 to 3.74, *n* = 20).

### Risk of Bias

The overall risk of bias was moderate.

**Figure 3:**
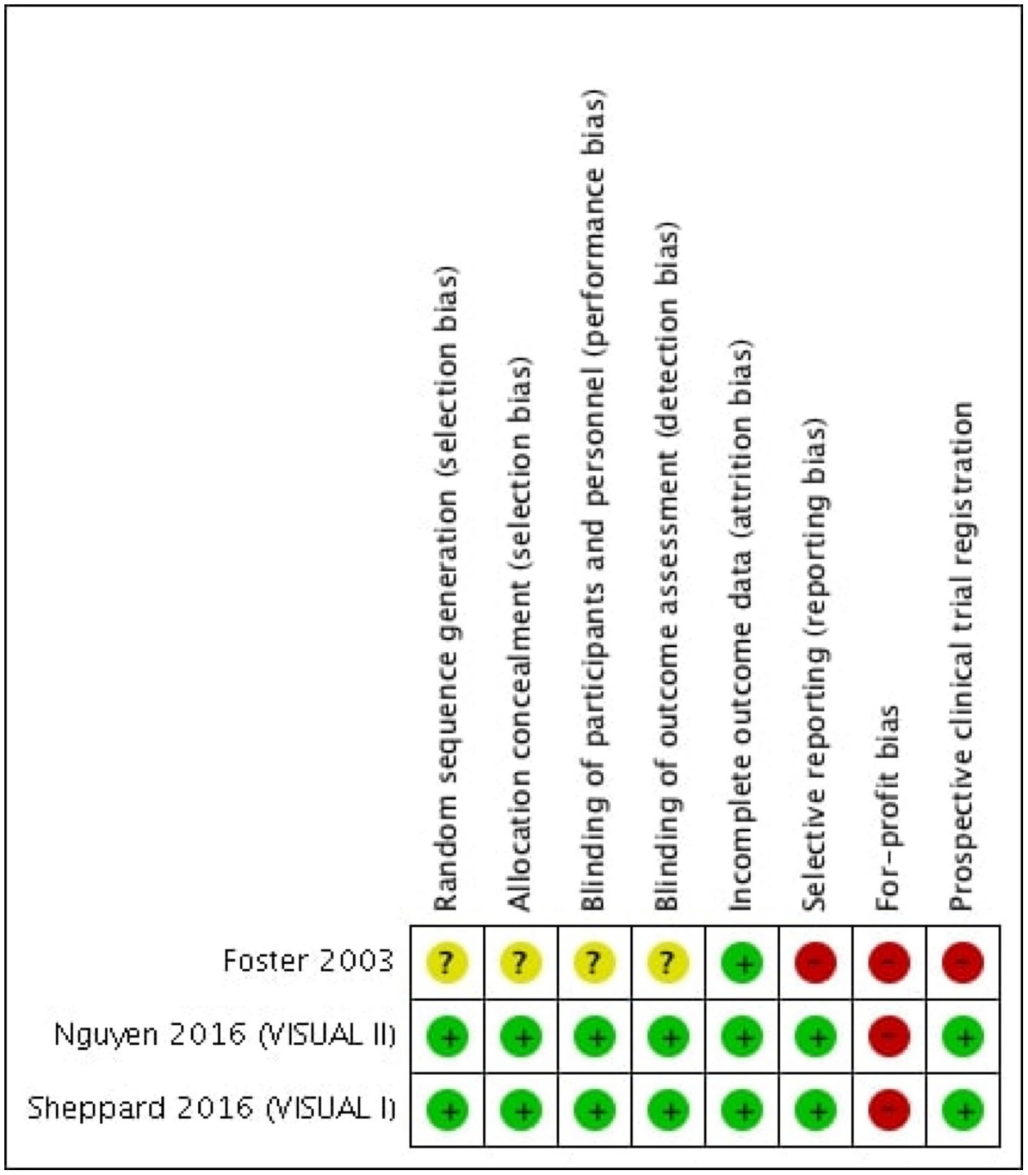
Risk of bias summary.

Incomplete outcome data (attrition bias) was the only item considered of low risk across all studies (Figure 3). In fact, in VISUAL I and II, missing outcome data is balanced in numbers across intervention groups and in Foster et al. (24) there is no missing data.

In VISUAL I and II, random sequence generation was considered of low risk because the investigators describe a random component in the sequence generation; allocation concealment (selection bias) was considered of low risk, since there was a computer-generated assignment sequence of allocation; blinding of participants, personnel and outcome assessors were also of low risk because measures to blind participants, personnel and outcome assessors from knowledge of which intervention a participant received are described; as study protocols are available and all studies' pre-specified outcomes were reported in the studies' main paper or in available Supplementary Files, selective reporting were considered of low-risk. Prospective trial registration was accomplished in both studies.

No available information was found regarding random sequence generation, allocation concealment (selection bias), blinding of participants and personnel or blinding outcome assessment in Foster et al. (24) and, therefore, these items were considered of unclear risk. Due to the lack of outcomes reported in ocular inflammation studies (change in BCVA in logMar, anterior chamber and vitreous inflammation grade) and lack of available protocol, selective reporting was considered of high risk in Foster et al. (24). Prospective clinical trials registration was not available in Foster et al. (24).

VISUAL I and II were industry-funded and in Foster et al. (24) the sponsor provided placebo and the active comparator; for these reasons, for-profit bias was considered of high risk across studies.

#### Risk of Bias Across Studies

Not performed due to lack information.

#### Additional Analysis

Not performed due to lack information.

## Discussion

### Summary of Main Findings

According with the GRADE, there is very low quality evidence that the risk of etanercept not worsening visual acuity in inactive NIU is not different from placebo, and high quality evidence that adalimumab increases this risk of not worsening visual acuity in active and inactive NIU. Furthermore, moderate quality evidence suggests that anti-TNF agents are not different from placebo on the risk of trial withdrawal (Supplementary Table 2). These conclusions are based on 2 randomized controlled trials enrolling 443 patients with active and inactive NIU, comparing adalimumab with placebo. Another small study with 20 patients with controlled NIU, failed to show a positive effect of etanercept vs. placebo in preventing uveitis flare.

Clinical, methodological and design heterogeneity between studies precluded a pooled analysis of efficacy outcomes. However, for safety, as we analyzed drug class-related adverse events common to all anti-TNF drugs, the results were pooled and the risk of adverse events did not differ between the anti-TNF and control arms. Of note, included studies were not adequately powered to study adverse events.

The use of two different drugs in the active arm [etanercept in Foster et al. (24) and adalimumab in VISUAL I and VISUAL II] and important disparities regarding the number of patients randomized [217 in VISUAL I, 226 in VISUAL II and 20 in Foster et al. (24)] are probably pivotal factors that explain the difference between risk of not worsening BCVA with anti-TNF and placebo in the different studies. Thus, results of this specific endpoint favored adalimumab over placebo in both VISUAL studies and suggested a worse efficacy profile in the etanercept over placebo, although this result was not statistically significant. Moreover, regarding population disparities, both VISUAL I and II focused ocular inflammation in the intermediate or posterior eye compartments or affecting the three eye compartments (panuveitis), whereas Foster et al. (24) included patients whose etiology of uveitis is more closely linked to anterior inflammatory processes (although the affected eye compartments is an item not specified in the study), such as HLA-B27-associated uveitis and arthritis-associated uveitis (29). It should be emphasized that Foster et al. (24) is a much less robust trial than the two VISUAL studies.

Adalimumab is a fully human-derived monoclonal antibody, produced by recombinant DNA technology. It binds to human TNF with high affinity and specificity, and blocks TNF pathway via interaction with p55 and p75 cell-surface TNF receptors (11, 30). Before these two studies with adalimumab, there was evidence of its efficacy and safety in adult NIU from case reports with adalimumab (31, 32), case series with adalimumab given for ocular disease (33–35), case series with adalimumab given for other rheumatic conditions (36), and prospective open-label trials (37–40). The cumulative evidence in literature of the effectiveness and safety of adalimumab in NIU together with its high specificity for TNF are in line with the favorable results observed in both VISUAL-I and II (41). These are well-designed multi-centric double-masked randomized trials that provide high-quality evidence for the use of adalimumab in active and non-inactive NIU. It should be noted though, that both of these studies excluded anterior NIU, for which high quality evidence is still lacking (42).

On the other hand, etanercept is a humanized, recombinant fusion protein, consisting of the p75 TNF receptor II, combined with the Fc tail domain of the human immunoglobulin (IgG) 1. This fusion protein blocks the interaction of TNF with cell surface TNF receptors (11). Initial studies suggested encouraging results for etanercept in ocular inflammation control (43). However, long term non-RCT follow-up studies and more recent studies proposed clear inferiority in ocular inflammation control compared to other anti-TNF, such as infliximab or adalimumab (44, 45). Furthermore, some authors also suggest that etanercept may have immunodysregulatory and even pro-inflammatory effects, triggering uveitis (39, 46, 47). The pathophysiology of this phenomenon is still ill-defined (48, 49). The only study included using etanercept suggested a decreased likelihood of VA preservation comparing to placebo, although not significant. Foster et al. (24)'s small sample size could have also contributed to the mentioned efficacy result. Another possible reason for these results could be the poor effect of etanercept in protecting against uveitis. It should be emphasized that Foster et al. published this trial in 2003 and only a few years later there was increasing evidence for the possible uveitis-inducing effect of this drug (43). In general, this trial was much smaller than the trials using adalimumab and the risk of bias was higher due to methodological reasons. Therefore, caution must be taken when considering its results.

Until now, only three RCT studying anti-TNF against placebo in NIU have been published, one with etanercept and two with adalimumab. Only one among the three available RCT was performed on patients with active NIU. The total number of patients so far treated with etanercept is too small for us to be confident to draw conclusions of its efficacy.

A next step for future trials would be the comparison vs. placebo of other anti-TNF drugs that are not approved for NIU management but are commonly used off-label, such as infliximab, or the comparison of anti-TNF drugs against conventional immunosupressors widely used in NIU. Also, considering trials evaluating not only the capability of anti-TNF to induce remission of the inflammatory process in patients with active NIU, but also assessing the capability of anti-TNF to prevent uveitic flare in patients with inactive NIU would be of paramount importance. Finally, given the limited number of RCT, the inclusion of observational studies would be a convenient approach to further study the safety and efficacy profile of these drugs in NIU.

### Limitations

Although study design across studies is identical (RCT evaluation anti-TNF vs. placebo in NIU), population and active interventions are not comparable across studies. The **three** studies have diverse methods, using distinctive drugs as active arm and report on widely different populations, which compromises data aggregation in the pooled-analysis for safety outcomes and efficacy comparisons. Secondly, although our initial aim was to evaluate efficacy and safety of anti-TNF in NIU, only **two** of **five** commercially available anti-TNF drugs were used in these **three** studies. Thirdly, the 2 largest trials included in this work have both been funded by the adalimumab drug company, which may introduce bias in the study design, data collection, data analysis and interpretation. We think that independent (academic and/or investigator-initiated) trials are required with longer follow-up to be able to draw more solid conclusions.

It should be noted the limited generalizability of RCT when it comes to safety analysis. Because populations are generally not very big, follow-up times are not long enough to identify rare adverse effects and patients with high risk of adverse effects are often excluded, RCT are not the best tools to study safety outcomes. This should be taken into account when interpreting our safety analysis (50). Additionally, profit bias was considered of high risk across studies. Despite these limitations, we believe that our work aggregates the currently best available evidence regarding the use of anti-TNF vs. placebo in NIU.

## Conclusions

According with our predefined outcomes, adalimumab seems efficacious for the treatment of active non-anterior NIU and in preventing flare-ups from inactive non-anterior NIU; and that adalimumab and etanercept are safe in people with NIU. Overall, the evidence is not sufficiently robust to determine the comparative effectiveness of anti-TNF in NIU. Our work highlights the scarce evidence in the field and the need for future robust trials to conclude about efficacy and safety of anti-TNF drugs in NIU

## Data Availability

Availability of data and material: DOI 10.1001/archopht.121.4.437, DOI 10.1056/NEJMoa1509852, DOI 10.1016/S0140-6736(16)31339-3.

## Author Contributions

CN, JC, and JF were the guarantors. FR, IL, DS, VR, and GD contributed to the concept and design, data acquisition, data analysis and interpretation of the data. Search strategies were designed, tested, and applied to databases by FR. IL and FR wrote the first draft of the manuscript. DS, FR, IL, VR, GD, CN, JC, and JF critically revised the manuscript. IL, FR, DS, VR, GD, CN, JC, and JF gave final approval of the submitted manuscript.

### Conflict of Interest Statement

The authors declare that the research was conducted in the absence of any commercial or financial relationships that could be construed as a potential conflict of interest.
