# Supplementary material for: Anti-TNF Drugs for Chronic Uveitis in Adults—A Systematic Review and Meta-Analysis of Randomized Controlled Trials
Source: Front Med (Lausanne). 2019 May 24;6:104. doi: 10.3389/fmed.2019.00104 (PMC6543521; doi:10.3389/fmed.2019.00104)
Supplement: Supplementary file 1 [file Table_1.pdf]

Supplementary Table 1: Population baseline characteristics by individual study

		Foster		VISUAL I		VISUAL II	
		Placebo	Anti-TNF	Placebo	Anti-TNF	Placebo	Anti-TNF
No. of patients		10	10	107	110	111	115
Age in years mean (SD)		45 (N.S.)	48 (N.S.)	42.6 (14.2)	42.7 (15.6)	42.2 (14.0)	42.9 (12.9)
Male gender (n/)%		1/10%	73/30%	a 93/43%*		39/35%	49/43%
Race (n/%)	White	N.S.		86/80%	88/80%	93/84%	96/83%
	Black			12/11%	11/10%	8/7%	6/5%
	Asian			2/2%	4/4%	3/3%	3/3%
	Other			7/7%	7/6%	5/5%	9/8%
Type of uveitis (n/%)	Panuveitis	N.S.		47/44%	50/45%	46/41%	57/50%
	Intermediate and posterior			0		1/1%	2/2%
	Intermediate			23/21%	24/22%	30/27%	17/15%
	Posterior			37/35%	36/33%	34/31%	39/34%
Diagnosis	Idiopathic uveitis	7/70%	5/50%	45/42%	36/33%	40/36%	29/25%
	Birdshot choroidopathy	0	0	20/19%	24/22%	15/14%	15/13%
	Multifocal choroiditis and panuveitis	0	0	3/3%	8/7%	2/2%	5/4%
	Vogt-Koyanagi-Harada	0	0	14/13%	11/10%	25/23%	26/23%
	Behçet's	0	0	4/4%	12/11%	6/5%	18/16%
	Sarcoid	0	0	8/7%	10/9%	14/13%	10/9%
	HLA-B27-associated	1/10%	2/20%	0	0	0	0
	SLE-associated	1/10%	2/20%	0	0	0	0
	Arthritis-associated	1/10%	1/10%	0	0	0	0
	Other	0	0	13/12%	9/8%	9/8%	12/10%
Duration of uveitis in months Mean (SD)		N.S.		51.0 (72.2)	40.2 (51.2)	51.0 (72.2)	40.2 (51.2)
Concomitant immunomodulatory treatment (n/%)	Azathioprine	All patients were under MTX at the beginning of the study, then suspended		4/4%	4/4%	11/10%	3/3%
	Cyclosporine			3/3%	10/9%	11/10%	15/13%
	Methotrexate			12/11%	9/8%	14/13%	19/17%

	Mycophenolate mofetil		14/14%	11/10%	17/15%	17/15%
Affected eye	Left only	N.S.	5/5%	5/5%	3/3%	2/2%
	Right only		3/3%	7/6%	4/4%	1/1%
	Both		99/93%	98/89%	104/94%	112/97%

Legend: No: number; HLA: human leucocyte antigen; TNF: tumor necrosis factor; N.S.: non-stated; MTX: methotrexate
* Only aggregated data for gender provided.
