# Supplementary material for: Anti-TNF Drugs for Chronic Uveitis in Adults—A Systematic Review and Meta-Analysis of Randomized Controlled Trials
Source: Front Med (Lausanne). 2019 May 24;6:104. doi: 10.3389/fmed.2019.00104 (PMC6543521; doi:10.3389/fmed.2019.00104)
Supplement: Supplementary file 2 [file Table_2.pdf]

Supplementary Table 2: Summary of findings

**Etanercept compared to placebo for inactive non-infectious uveitis**					
		**Anticipated absolute effects (95% CI)**			
		**Without Etanercept**	**With Etanercept**	**Difference**	
Risk of not worsening BCVA<br>№ of participants: 20 (1 RCT)	RR 0.90 (0.69 to 1.18)	100.0%	**90.0%** (69.0 to 100.0)	**10.0% fewer** (31 fewer to 18 more)	⊕ VERY LOW [a,b]
**Adalimumab compared to placebo for active non-infectious uveitis**					
		**Anticipated absolute effects (95% CI)**			
		**Without Adalimumab**	**With Adalimumab**	**Difference**	
Risk of not worsening BCVA<br>№ of participants: 217 (1 RCT)	RR 1.75 (1.32 to 2.32)	37.4%	**65.4%** (49.3 to 86.7)	**28.0% more** (12 more to 49.3 more)	⊕⊕⊕⊕ HIGH
**Adalimumab compared to placebo for inactive non-infectious uveitis**					
		**Anticipated absolute effects (95% CI)**			
		**Without Adalimumab**	**With Adalimumab**	**Difference**	
Risk of not worsening BCVA<br>№ of participants: 226 (1 RCT)	RR 1.31 (1.12 to 1.53)	65.8%	**86.2%** (73.7 to 100.0)	**20.4% more** (7.9 more to 34.9 more)	⊕⊕⊕⊕ HIGH
**Anti-TNF compared to placebo for non-infectious uveitis**					
		**Anticipated absolute effects (95% CI)**			
		**Without Anti-TNF**	**With Anti-TNF**	**Difference**	
Risk of withdrawals<br>№ of participants: 472 (3 RCTs)	RR 1.63 (0.62 to 4.26)	9.7%	**15.9%** (6.0 to 41.5)	**6.1% more** (3.7 fewer to 31.8 more)	⊕⊕⊕ MODERATE [b,c]

GRADE Working Group grades of evidence: **High quality:** We are very confident that the true effect lies close to that of the estimate of the effect; **Moderate quality:** We are moderately confident in the effect estimate: The true effect is likely to be close to the estimate of the effect, but there is a possibility that it is substantially different; **Low quality:** Our confidence in the effect estimate is limited: The true effect may be substantially different from the estimate of the effect; **Very low quality:** We have very little confidence in the effect estimate: The true effect is likely to be substantially different from the estimate of effect . ***The risk in the intervention group** (and its 95% confidence interval) is based on the

assumed risk in the comparison group and the **relative effect** of the intervention (and its 95% CI). a. Very serious risk of bias due to multiple domains at high or unclear risk; b. Optimal information size not met; c. Although one trial was at high risk of bias, it represented less than 10% of information in the analysis. As so we opted not to downgrade. **CI:** Confidence interval; **RR:** Risk ratio
