# Supplementary material for: Anti-TNF Drugs for Chronic Uveitis in Adults—A Systematic Review and Meta-Analysis of Randomized Controlled Trials
Source: Front Med (Lausanne). 2019 May 24;6:104. doi: 10.3389/fmed.2019.00104 (PMC6543521; doi:10.3389/fmed.2019.00104)
Supplement: Supplementary file 3 [file Data_Sheet_1.pdf]

Data supplements

Appendix 1

Protocol and registration:

The protocol followed Preferred Reporting Items of Systematic reviews and Meta Analyses Protocols (PRISMA-P) guidelines[24] and it was registered at Prospero database (CRD42016039068). For the purpose of this systematic review we followed PRISMA guidelines.[25] Statistical data reporting followed the Statistical Analyses and Methods in the Published Literature (SAMPL) guidelines.[26]

Eligibility criteria:

The following criteria were used for selecting studies for this review:

We analysed parallel RCTs of any duration, assessing efficacy and safety of anti-TNF versus control interventions in patients with chronic NIU. Non-parallel study designs were excluded. The control arm was defined as patients not receiving anti-TNF, including but not limited to placebo interventions. All administration schedules and typologies were accepted.

Regarding participants, RCTs had to include adult patients aged above 18 years old with a clinical diagnosis of chronic NIU, irrespective of the aetiology. Chronic NIU was defined as inflammation of any part of the uvea (choroid, ciliary body and/or iris) that lasts for 12 or more weeks, after an infectious aetiology has been excluded, or if there is a high suspicion of an immune-mediated underlying mechanism, which may occur isolated or in association with a systemic condition.[1,2,18,23,27] There were no restrictions regarding the number of participants recruited to trials or the number of recruitment centres.

Information sources:

We searched MEDLINE (from inception to January 2019), EMBASE (from inception to December 2018), Cochrane Controlled Trials Register (CENTRAL) (The Cochrane Library, October 2018), OpenSIGLE.inist.fr (from inception to December 2018), NTIS.gov (from inception to December 2018), ClinicalTrials.gov (from inception to January 2019), ClinicalTrialsRegistry.eu (from inception to January 2019). Grey literature was retrieved from appropriate databases (www.opensigle.inist.fr; www.ntis.gov). Clinical trials registries were pursued (www.clinicaltrial.gov; www.clinicaltrialsregistry.eu). Non-English papers were equally assessed, translated as necessary and evaluated for inclusion. The search strategy also included: search through reference lists of located trials; hand search of abstracts of international congresses of ophthalmology and rheumatology, e.g. the American Academy of Ophthalmology annual meetings, the European Society of Ophthalmology congresses, the Association for Research in Vision and Ophthalmology annual meetings, the European Society of Retina Specialists meetings, the International Ocular Inflammation Society meetings, the Annual European Congress of Rheumatology (from 2011 to 2018) and the American College of Rheumatology Annual Meeting (from 2011 to 2018); personal communication with other researchers in the field; whenever necessary, authors of published trials were contacted for further information and unpublished data.

Search:

For the identification of studies considered for inclusion in this review, detailed search strategies were developed for each database explored. Please refer to Appendix A for the MEDLINE search strategy, Appendix B for the EMBASE search strategy, and Appendix C for the CENTRAL strategy.

Study selection:

Two independent review authors (IL, DS) assessed if the studies identified by the search strategy were illegible, read each of the titles and abstracts of the reports. If there were no

abstract, the report was retrieved in full text. The same authors independently screened the full-texts of potentially illegible studies. Disagreements were resolved by discussion and consensus was reached with the participation of three authors (IL, DS and FBR).

Data collection process:

Two review authors (IL, DS) independently assessed the full-text articles of included studies for methodological quality and data extraction, then extracted the data onto standardized forms and crosschecked them for accuracy. Disagreements were resolved by discussion and consensus was reached with participation of three authors (DS, IL and FBR).

Data items:

The following data were extracted from each study: demographics and clinical baseline imbalances, number of withdrawals, loss to follow-up, if any; full description of intervention, duration of treatment period and follow-up, number of randomised participants to each arm, compliance and dropouts, reasons for dropouts, and ability to perform an intention-to-treat analysis; definition of outcomes, use of validated measurement tools, time-point measurements, change from baseline or post-interventional measures, and missing outcomes, if any.

Risk of bias in individual studies:

The recommended Cochrane Collaboration's tool for assessing risk of bias was used in this review, which targets six specific risk of bias domains (i.e. sequence generation, allocation concealment, blinding of participants and personnel, blinding of outcome assessment, incomplete outcome data and selective outcome reporting).[28] Two additional domains were added: for-profit bias, and prospective trial registration. Two independent review authors (IL, DS) performed critical assessments for each domain. Disagreements were resolved by discussion and, if needed, consensus was reached with the participation of a third author (FBR).

Summary measures:

The primary efficacy outcome was preservation of best-corrected visual acuity (BCVA) (logMAR) measured and presented according to the standard procedure developed for the Early Treatment Diabetic Retinopathy Study[29] (mean ± standard deviation) in the end of the study.

The primary safety outcome was withdrawals due to adverse events.

The secondary efficacy outcomes were: change from baseline in anterior chamber and/or vitreous inflammation grade and (according to Standardization of Uveitis Nomenclature (SUN) [5,30] recorder in the last measurement, reported in mean ± standard deviation. In case one eye improved but the other eye deteriorate, we considered the changes from baseline in anterior chamber and/or vitreous inflammation reported in the worsening eye; other secondary outcomes were median time to Optical Coherence Tomography (OCT), evidence of cystoid macular oedema (CME), change from baseline in the score (mean ± standard deviation) after 16-20 weeks of therapy obtained in a vision-specific questionnaire, the National Eye Institute Visual Functioning Questionnaire (NEI-VFQ) 25[31].

The secondary safety outcomes were: number of patients with infections, number of patients with new onset or reactivated tuberculosis, number of patients with injection site or allergic reactions, number of patients with immunogenicity related adverse events and number of patients with other adverse events.

For dichotomous outcomes, we retrieved the number of included patients in each treatment arm – anti-TNF and control – and the number of patients experiencing the outcome. We reported numerators and denominators for all percentages. For continuous outcomes, we retrieved the mean or median – the latter converted to mean using statistically validated

methods.[32] We reported means and standard deviations. For counts, we reported the mean per participant and standard deviations as presented by the authors. We carried an intention-to-treat analysis using risk ratios for dichotomous outcomes and mean differences for continuous outcomes and rates and standard deviations for counts. The proportion of patients with adverse events was compared between treatment arms, and further analysis was performed in the most frequent complications reported in trials. Data was pooled from the studies where adequate, and used for comparison.

Synthesis of results:

Statistical analysis was performed with Review Manager version 5.3. Dichotomous data were preferentially reported in this review as risk ratios using a Mantel-Haenszel fixed-effects (FE) model and 95% confidence intervals (95% CI). When no risk estimate was available, the crude RR was derived from raw data. Continuous outcomes, such as efficacy measures, were reported as mean differences and 95% CI. For counts, the rate ratios were pooled using the generic inverse-variance method. Whenever the number of events detected was equal to zero, we applied a correction factor of 0.5 to avoid computational errors.[28] Where data from the studies reports could not be combined into a meta-analysis, a narrative approach to synthesis of the results was included in the review text.

Heterogeneity between trial results was tested using an I^2 statistic to quantify inconsistency across studies. When considerable heterogeneity was present (i.e. $I^2 > 50\%$), the possible causes of heterogeneity were explored by conducting subgroup analysis. Where heterogeneity could not readily be explained by the exploratory analyses performed, it was incorporated into a Random-Effects (RE) meta-analysis model.

Risk of bias across studies:

Publication bias was planed to be assessed through visual inspection of funnel plots asymmetry and Peters' regression tests[33,34], if more than 10 studies per outcome were available.[28]Unfortunately we did not have enough power to perform theses analyses.

Additional analysis:

Subgroup analysis was pre-planned for the following: uveitis etiology (primary versus secondary; different secondary causes), risk of bias, pharmacological compound (infliximab versus adalimumab versus certolizumab versus golimumab versus etanercept), age of participants, mean follow-up time, steroid-resistant or dependent, and BCVA.[28] Unfortunately, we did not have enough power to perform theses analyses.

Confidence in cumulative evidence:

All primary outcomes were evaluated according to quality of evidence using the Grading of Recommendations Assessment, Development and Evaluation (GRADE) working group methodology.[35] Moreover, the secondary safety outcomes and the proportion of patients achieving an excellent functional outcome at 90 days after randomization were graded. The following domains were assessed: risk of bias, consistency, directness, precision and publication bias; when suitable, further domains were evaluated. Quality of evidence fell under one of the following categories: high (i.e.: further research is very unlikely to change the level of confidence in the estimate of effect), moderate (i.e.: further research is likely to have an important impact on the level of confidence in the estimate of effect and may change the estimate), low (i.e.: further research is very likely to have an important impact on the level of confidence in the estimate of effect and is likely to change the estimate), and very low (i.e.: very uncertain about the estimate of effect).

Appendix 2: MEDLINE search strategy

1. exp Uveitis/
2. uveiti*.tw.
3. exp Panuveitis/
4. Panuveitis.tw.
5. exp Pars Planitis/
6. Pars Planitis.tw.
7. exp Panophthalmitis/
8. Panophthalmiti*.tw.
9. exp Iridocyclitis/
10. (Iridocycliti* or Heterochromic Cycliti* or “anterior scleritis”).tw.
11. exp Iritis/
12. Iriti*.tw.
13. exp Choroiditis/
14. (choroiditi* or retinochoroiditi* or chorioretinitis).tw.
15. (uveoretinitis or uveo retinitis).tw.
16. vitritis*.tw.
17. exp Retinitis/
18. (retinitis or neuroretinitis).tw.
19. or/1-18
20. exp Receptors, Tumor Necrosis Factor/
21. exp Tumor Necrosis Factor-alpha/
22. exp Antibodies, Monoclonal/
23. anti-tumo?r necrosis factor$.mp
24. anti-TNF.mp
25. etanercept.mp
26. infliximab.mp
27. adalimumab.mp
28. golimumab.mp
29. certolizumab.mp
30. or/20-29
31. randomized controlled trial.pt.
32. controlled clinical trial.pt.
33. randomized.ab.
34. placebo.ab.
35. drug therapy.fs.
36. randomly.ab.
37. trial.ab.
38. groups.ab.
39. or/31-38
40. and/19,30,39
41. exp animals/ not humans.sh.
42. 40 not 41

Appendix 3: EMBASE strategy

1. exp uveitis/
2. “uveiti*”.ab,ti.
3. exp autoimmune uveitis/
4. choroiditi*.ab,ti.
5. chorioiditi*.ab,ti.
6. exp chorioretinitis/
7. retinochoroiditi*.ab,ti.
8. chorioretiniti*.ab,ti.
9. exp intermediate uveitis/
10. pars planitis.ab,ti.
11. exp iridocyclitis/

12. iridocycliti*.ab,ti.
13. scleritis.ab,ti.
14. exp iritis/
15. iriti*.ab,ti.
16. exp uveoretinitis/
17. uveoretinitis.ab,ti.
18. uveo retinitis.ab,ti.
19. exp vitritis/
20. vitritis*.ab,ti.
21. Panuveitis.ab,ti.
22. panophthalmiti*.ab,ti.
23. exp retinitis/
24. retinitis.ab,ti.
25. Neuroretinitis.ab,ti.
26. or/1-25
27. exp tumour necrosis factor alpha antibody/
28. tumour necrosis factor alpha antibody.ab,ti.
29. exp tumor necrosis factor antibody/
30. tumor necrosis factor antibody.ab,ti.
31. exp tumour necrosis factor/
32. anti tumour necrosis factor.ab,ti.
33. exp tumor necrosis factor alpha/
34. tumour necrosis factor alpha.ab,ti.
35. anti tumor necrosis factor alpha.ab,ti.
36. anti TNF.ti,ab.
37. TNF alpha antibody.ti,ab.
38. exp etanercept/
39. etanercept.ti,ab.
40. exp infliximab/
41. infliximab.ti,ab
42. exp adalimumab/
43. adalimumab.ti,ab.
44. exp golimumab/
45. golimumab.ti,ab
46. exp certolizumab pegol/
47. certolizumab.ti,ab.
48. or/27-47
49. (random$ or placebo$ or single blind$ or double blind$ or triple blind$).ti,ab.
50. RETRACTED ARTICLE/
51. or/49-50
52. (animal$ not human$).sh,hw.
53. (book or conference paper or editorial or letter or review).pt. not exp randomized controlled trial/
54. (random sampl$ or random digit$ or random effect$ or random survey or random regression).ti,ab. not exp randomized controlled trial/
55. or/52-54
56. 51 not 55
57. and/26,48,56

Appendix 4: CENTRAL strategy

1. MeSH descriptor: [Uveitis] explode all trees
2. uveiti*
3. MeSH descriptor: [Panuveitis] explode all trees
4. panuveiti*
5. MeSH descriptor: [Pars Planitis] explode all trees
6. Pars Planitis
7. MeSH descriptor: [Panophthalmitis] explode all trees

8. Panophthalmiti*
9. MeSH descriptor: [Iridocyclitis] explode all trees
10. (Iridocycliti* or Heterochromic Cycliti* or anterior scleritis)
11. MeSH descriptor: [Iritis] explode all trees
12. Iriti*
13. Choroiditis
14. (choroiditi* or retinochoroiditi* or chorioretinitis)
15. (uveoretinitis or uveo retinitis)
16. vitritis*
17. MeSH descriptor: [Retinitis] explode all trees
18. (retinitis or neuroretinitis)
19. #1 or #2 or #3 or #4 or #5 or #6 or #7 or #8 or #9 or #10 or #11 or #12 or #13 or #14 or #15 or #16 or #17 or #19
20. MeSH descriptor: [receptors, tumor necrosis factor] explode all trees
21. receptors, tumor necrosis factor
22. MeSH descriptor: [tumor necrosis factor-alpha] explode all trees
23. tumor necrosis factor-alpha
24. MeSH descriptor [antibodies, monoclonal] explode all trees
25. antibodies, monoclonal
26. #20 OR #21 OR #22 OR #23 OR #24 OR #25
27. #19 AND #26

Appendix 5: OpenSIGLE strategy

(uveitis OR panuveitis OR choroiditis OR pars planitis OR panophthalmitis OR iridocyclitis OR iritis OR retinitis) AND (etanercept OR infliximab OR adalimumab OR golimumab OR certolizumab)

Appendix 6: NTIS strategy

(uveitis OR panuveitis OR choroiditis OR pars planitis OR panophthalmitis OR iridocyclitis OR iritis OR retinitis) AND (etanercept OR infliximab OR adalimumab OR golimumab OR certolizumab)

Appendix 7: ClinicalTrials strategy

(uveitis OR panuveitis OR choroiditis OR pars planitis OR panophthalmitis OR iridocyclitis OR iritis OR retinitis) AND (etanercept OR infliximab OR adalimumab OR golimumab OR certolizumab)

Appendix 8: ClinicalTrialsRegestry strategy

(uveitis OR panuveitis OR choroiditis OR pars planitis OR panophthalmitis OR iridocyclitis OR iritis OR retinitis) AND (etanercept OR infliximab OR adalimumab OR golimumab OR certolizumab
